# Jagged1 contained in MSC-derived small extracellular vesicles promotes squamous differentiation of cervical cancer by activating NOTCH pathway

**DOI:** 10.1007/s00432-023-05495-3

**Published:** 2023-11-23

**Authors:** Weizhao Li, Xunzhi Zhang, Tianshun Gao, Lixiang Liu, Chi Zhang, Huan Yang, Jiayuan Xie, Wei Pan, David Y. B. Deng, Changlin Zhang, Tian Li

**Affiliations:** 1 Department of Gynecology, Pelvic Floor Disorders Center, Scientific Research Center, The Seventh Affiliated Hospital of Sun Yat-Sen University, Shenzhen, China; 2 Shenzhen Key Laboratory of Chinese Medicine Active Substance Screening and Translational Research, Shenzhen, China; 3 College of Life Sciences and Oceanography, Shenzhen University, Shenzhen, China

**Keywords:** Mesenchymal stem cells, MSC-sEV, Differentiation therapy, Cervical cancer, NOTCH pathway

## Abstract

**Purpose:**

Cervical cancer is the fourth most common cancer in women and poses a major threat to women's health, urgently requiring new treatment methods.

**Methods:**

This study first successfully extracted and identified small extracellular vesicles secreted by human umbilical cord-derived mesenchymal stem cells. We studied the effects of MSC-sEV on the squamous differentiation levels of cervical cancer CaSki cells in vitro, and explored the effects of MSC-sEV on the NOTCH pathway, the growth, proliferation, migration abilities and squamous differentiation levels of cervical cancer cells. The roles of MSC-sEV were also verified in human keratinocyte HaCaT cells.

**Results:**

The results showed that Jagged1 protein on MSC-sEV can bind to NOTCH1 on cervical cancer cells, activate NOTCH signaling, and promote squamous differentiation levels in CaSki cells, thus inhibiting the growth, proliferation and migration abilities of CaSki cells. MSC-sEV can also activate the NOTCH pathway in HaCaT cells, but promote the viability of HaCaT cells.

**Conclusion:**

MSC-sEV can activate the NOTCH pathway to promote squamous differentiation of CaSki cells and inhibit the growth proliferation and migration abilities of CaSki cells which may be a new mechanism for cervical cancer treatment.

**Supplementary Information:**

The online version contains supplementary material available at 10.1007/s00432-023-05495-3.

## Introduction

Cervical cancer is a malignant tumor occurring in the cervix and is the fourth most common cancer among women worldwide (World Health Organization 2021). Cervical cancer is mainly caused by persistent infection with human papillomavirus (HPV), with HPV 16 and 18 types accounting for over 70% of cervical cancer cases (Cohen et al. 2019). HPV infects cervical basal layer cells through micro-lesions in the cervix. The E6 and E7 proteins encoded by HPV can activate the host cell DNA replication mechanism, allowing continuous viral DNA synthesis and generation of new HPV viruses (Yeo-Teh et al. 2018). HPV can also gradually integrate its genome into the host genome, leading to cervical intraepithelial neoplasia and eventually cervical cancer (Rodríguez et al. 2008).

The cervical tissue is composed of basal cells, spinous cells, granular cells and keratinized squamous epithelium from inside out (Gonzales and Fuchs 2017). Their squamous differentiation levels gradually increase, but their self-replication abilities gradually decline. Since HPV itself cannot encode enzymes and polymerases for self-replication, it can only infect actively proliferating cells (Crosbie et al. 2013). After HPV infection, it can damage cell squamous differentiation abilities and promote proliferation. HPV E6 and E7 proteins can also inhibit basal cell squamous differentiation by blocking NOTCH pathway activation (White 2019). Some studies have found that HPV E6 and E7 can inhibit squamous differentiation by regulating TGF β and calcium absorption (Chen et al. 1995; French et al. 2013).

Mesenchymal stem cell-derived extracellular vesicles (MSC-sEV) are a type of extracellular vesicle secreted by mesenchymal stem cells (MSC), usually less than 200 nm in diameter, and have shown potential in tumor treatment (Théry et al. 2018). The effects of MSC-sEV on cervical cancer are not yet fully understood. Burcin et al. found that MSC-sEV can promote apoptosis of cervical cancer HeLa cells and inhibit their epithelial-mesenchymal transition (EMT) (Abas et al. 2022). However, it remains unknown whether and how MSC-sEV affect the squamous differentiation abilities of cervical cancer cells.

In this study, we explored the effects of MSC-sEV on squamous differentiation and proliferative and migration abilities of cervical cancer CaSki cells, and investigated their effects on the NOTCH pathway and underlying molecular mechanisms. We also explored the effects of MSC-sEV on keratinocyte HaCaT cells. Our research provides new insights into the treatment of cervical cancer.

## Materials and methods

### Isolation and identification of MSC

We collected fresh umbilical cords from full-term cesarean section deliveries, and the mothers did not have gestational diabetes, infections, fever, or autoimmune diseases. The Ethical Committee of Sun Yat-sen University Affiliated Seventh Hospital approved the acquisition of umbilical cord specimens (KY-2022-060-01). All mothers were fully informed and gave their consent. The isolation and identification of MSC were executed as described before (Zhang et al. 2023).

### Isolation and identification of MSC-sEV

#### Isolation of MSC-sEV

We collected MSC-sEV from the conditioned medium of MSC using an ultracentrifugation method. When the confluence of MSC reached 70%-80%, we removed the supernatant and washed the cells three times with 1 × PBS to eliminate the influence of MSC-sEV from fetal bovine serum. Then, we added serum-free DMEM/F12 medium. After 48 h of cultivation, we collected the supernatant and immediately extracted the MSC-sEV or stored them at – 80 °C. The supernatant was centrifuged at 300*g* for 10 min, 2000*g* for 10 min, and 10,000*g* for 30 min. Subsequently, the supernatant was filtered using a 0.22 μm pore size membrane (SLGP033RB-0.22, Millipore, USA). The filtrate was further centrifuged at 120,000*g* for 70 min to collect the precipitate, which was then resuspended in DPBS. After another centrifugation at 120,000*g* for 70 min, the supernatant was removed, and the MSC-sEV were resuspended in 100μL DPBS to obtain highly pure and concentrated MSC-sEV. The concentration of MSC-sEV was measured by BCA assay according to the instruction book (E112-01, Vazyme, China).

#### Nanoparticle tracking analysis (NTA)

The particle size of MSC-sEV was measured using nanoparticle tracking analysis (NTA). MSC-sEV were diluted 100 times with 1 × DPBS, and the diluted solution was pumped into the NanoSight device (NS300, Malvern, UK), taking care to avoid the formation of bubbles. Each measurement lasted for 1 min, and a total of 5 measurements were conducted. The average value of the 5 measurements was calculated.

#### Transmission electron microscopy (TEM)

The morphology of MSC-sEV was observed using an electron microscope. A drop of 20μL MSC-sEV was placed on a carbon-coated copper grid and left for 3–5 min. Excess liquid was then removed using filter paper. Next, 2% phosphotungstic acid was added to the grid and left for 1–2 min. Excess liquid was again removed using filter paper, and the grid was air-dried at room temperature. The samples were observed and the images were collected and analyzed under a transmission electron microscope (HT7800, HITACHI, Japan).

### Western blot (WB)

Protein were detected using the standard Western blot method. MSC, CaSki, HaCaT cells and MSC-sEV were lysed using RIPA lysis buffer, and then the lysates were mixed with loading buffer and boiled for denaturation. SDS-PAGE was performed to separate proteins of different sizes, and the proteins were subsequently transferred onto a 0.22 μm PVDF membrane. Finally, the membrane was exposed using a chemiluminescence imaging system (ChemiDoc Imaging System, Bio-Rad, USA). The antibody used are provided in Supplementary Table 1.

### Cell culture

HPV 16&18 positive human cervical cancer cells CaSki and human immortalized keratinocytes HaCaT cells were obtained from ATCC (American Type Culture Collection). They were cultured in high glucose DMEM medium (Gibco, United States) supplemented with 10% fetal bovine serum (04–001-1ACS, BI, Israel), 100 U/mL penicillin, and 100 μg/mL streptomycin. The cells were maintained in a 37 °C incubator with 5% CO_2_.

### RNA isolation and real-time quantitative polymerase chain reaction (qPCR)

The mRNA expression levels of various genes were detected using the qPCR method. RNA was extracted from CaSki cells using the RNA rapid extraction kit (RN001, ES Science, China) according to the instructions. cDNA was obtained using the fast reverse transcription kit (RT001, ES Science, China). The detection was performed using SYBR qPCR Mix (Q311, Vazyme, China) in a Bio-Rad CFX96 instrument. The data were normalized using β-Actin as the internal reference, and the 2^^(−ΔΔCT)^ method was used for calculation. The primer sequences used are provided in Supplementary Table 2.

### CaSki cells uptake dil labeled MSC-sEV

MSC-sEV were labeled with Dil dye to investigate their ability to enter CaSki cells. 1 mg/mL Dil dye solution (dissolved in DMSO) (D3911, ThermoFisher) was mixed with 500 μg/mL MSC-sEV at a ratio of 1:50. The mixture was incubated at room temperature, protected from light, for 30 min to allow the Dil dye to incorporate into the membrane structure of MSC-sEV, facilitating the observation of their location.

CaSki cells were seeded in a 12-well plate with a density of 20,000 cells per well. After the cells adhered to the plate, the Dil-labeled MSC-sEV (Dil-MSC-sEV) were added. After 24 h, the cells were observed and photographed under a fluorescence microscope. If a fluorescent signal of Dil was observed on the surface of CaSki cells, it indicates that the Dil-labeled MSC-sEV were taken up by the CaSki cells.

### Cell counting kit-8 (CCK8)

Cell viability was measured using the CCK8 assay. In a 96-well cell culture plate, 10,000 CaSki cells were added to each well. After the cells adhered to the plate, an equal volume of MSC-sEV or DPBS (control) was added. After 24 h, the supernatant was removed, and 100 μL of CCK8 working solution (CCK8: serum-free 1640 medium = 1:10) was added to each well. The plate was then placed in a 37 °C incubator and incubated for 1 h. The absorbance at 450 nm wavelength was measured with a microplate reader (Synergy H1M, BioTek, USA). Each group was conducted three times, respectively.

### Clonogenic assay

The clonogenic assay was performed to assess the effect of MSC-sEV on the proliferative capacity of CaSki cells. In a 6-well plate, 1,000 CaSki cells were added to each well, along with the corresponding concentration of MSC-sEV. The culture medium was changed every 3 days. After 10 to 14 days of incubation, the culture was terminated. The supernatant was discarded, and the cells were fixed with methanol for 15 min. Subsequently, they were stained with 0.1% crystal violet solution for 15 min. The cells were then washed, air-dried, and photographed. Cell clusters containing more than 50 cells were counted to assess the impact of MSC-sEV on the clonogenic potential of CaSki cells. Each group was conducted three times, respectively.

### Transwell

The Transwell assay was used to assess the effect of MSC-sEV on the invasive ability of CaSki cells. Firstly, a suspension of CaSki cells in serum-free DMEM medium was prepared. The suspension was added to the Transwell chamber, with a volume of 200 μL, containing a total of 50,000 CaSki cells. In the lower chamber, 500 μL of complete DMEM medium (containing 10% fetal bovine serum) was added.

After 24 h of incubation, the culture was terminated. The cells were fixed with methanol solution for 15 min. Subsequently, they were stained with 0.1% crystal violet solution for 15 min. The cells were then washed, and the non-invasive cells on the upper side of the transwell chamber were gently wiped away. The chamber was air-dried, and photographs were taken. Select 5 views randomly, and the average cell count was calculated. The number of invasive cells was counted to evaluate the impact of MSC-sEV on the migration ability of CaSki cells. Each group was conducted three times, respectively.

### Statistical analysis

The results were reported as the mean ± standard deviation. The statistical analyses for each figure are provided in their respective legends. The student’s *t*-test was employed for two-group comparisons, while ANOVA analysis was used for three-group comparisons. The GraphPad Prism 9.0 software was utilized to calculate the statistical p-values using the appropriate tests. A significance level of *p* < 0.05 was considered statistically significant (ns: no significance, *p < 0.05, **p < 0.01, ***p < 0.001, ****p < 0.0001).

## Results

### Isolation and identification of hucMSC

Based on the International Society for Cellular Therapy (ISCT) criteria (Dominici et al. 2006), we confirmed that human umbilical cord mesenchymal stem cells (hucMSC) isolated exhibited adherence and differentiation capabilities. Positive and negative cell surface markers expressed by hucMSC met the requirements.

Primary hucMSC were obtained via tissue culture. Umbilical cord Wharton's jelly fragments were cultured in a flask, resulting in fibroblast-like adherent cells within 7–10 days, indicating the presence of MSC (Supplementary Fig. 1A). Osteogenic differentiation medium induced calcium deposition, confirmed by Alizarin Red S staining (Supplementary Fig. 1B). Adipogenic differentiation medium led to the presence of lipid droplets within cells, verified by Oil Red O staining (Supplementary Fig. 1C).

Flow cytometry analyzed cell surface marker expression on MSC. Positive markers for MSC (CD73, CD90, and CD105) exhibited expression rates above 95% (Supplementary Fig. 1D–F). Conversely, negative markers for MSC (HLA-DR, CD11b, CD19, CD34, and CD45), showed expression rates below 2% (Supplementary Fig. 1G–K).

### Identification of MSC-sEV

**Fig. 1:**
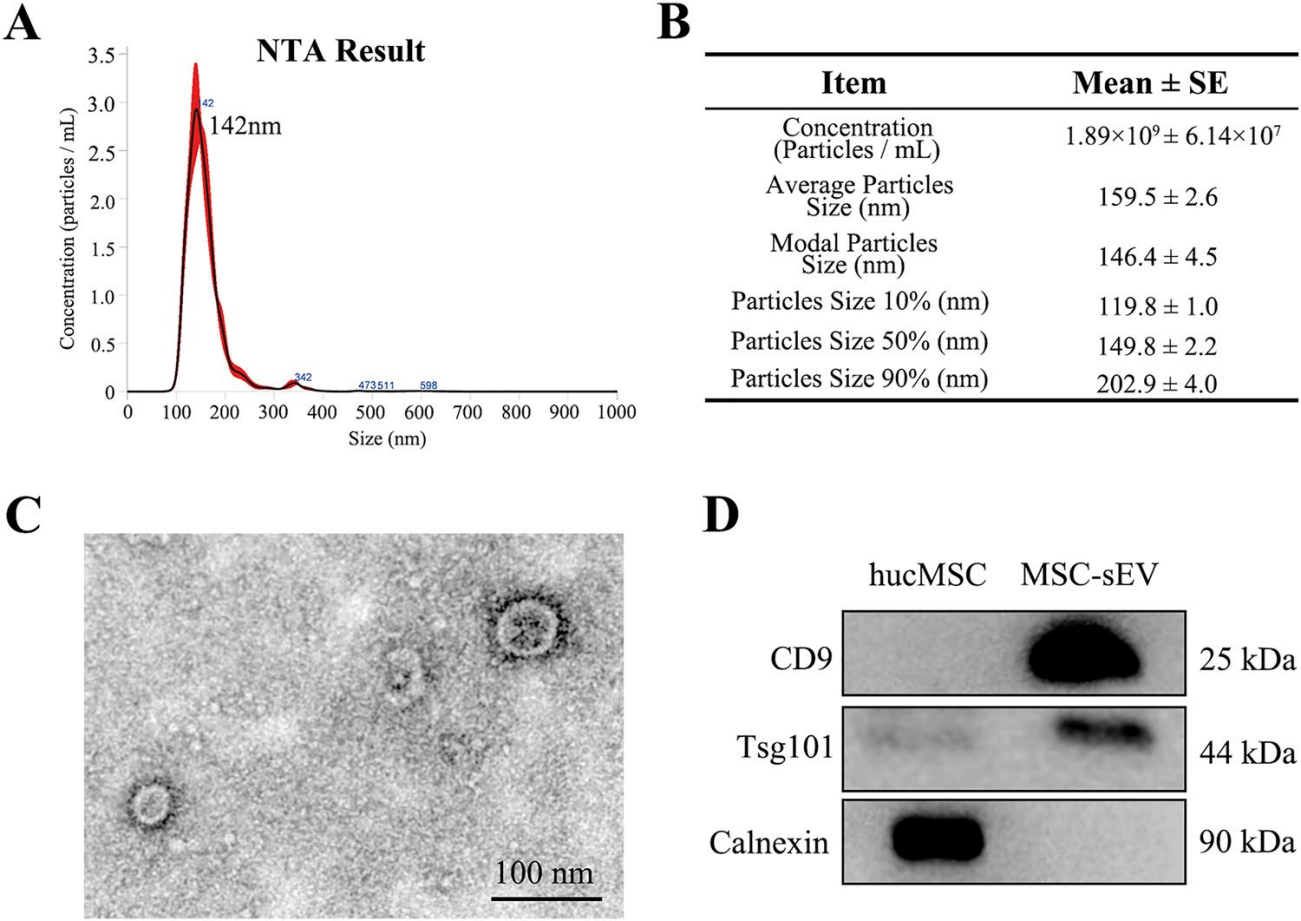
Identification of MSC-sEV. **A** Nanoparticle tracking analysis showed the particle diameter distribution of MSC-sEV. **B** A table for the concentration and size of MSC-sEV. (C) Transmission electron micrographs of MSC-Sev (Scale bar, 100 nm). (D) Western Blot showed the positive protein marker (CD9 and Tsg101) and negative protein marker (Calnexin)

To confirm if the extracted MSC-sEV meet international standards (Théry et al. 2018), we assessed their size, morphology, and positive and negative protein markers. NTA measured the MSC-sEV size, with a peak diameter of 142 nm (Fig. 1A). The average size of MSC-sEV was 159.5 nm, complying with international standards (Fig. 1B). TEM displayed double-concave disc-shaped vesicular structures, indicating excellent morphological quality of MSC-sEV (Fig. 1C). Western blot detected positive membrane protein markers CD81 and cytosolic protein marker Tsg101, while the negative protein marker Calnexin was absent in MSC-sEV (Fig. 1D). In summary, our MSC-sEV demonstrate high quality and meet international standards.

### MSC-sEV inhibited cell viability, clonogenic and migration of CaSki

To determine MSC-sEV effects on cervical cancer CaSki cells, we assessed MSC-sEV uptaked by CaSki cells and evaluated cell viability, colony formation, and invasion in response to different MSC-sEV concentrations.

**Fig. 2:**
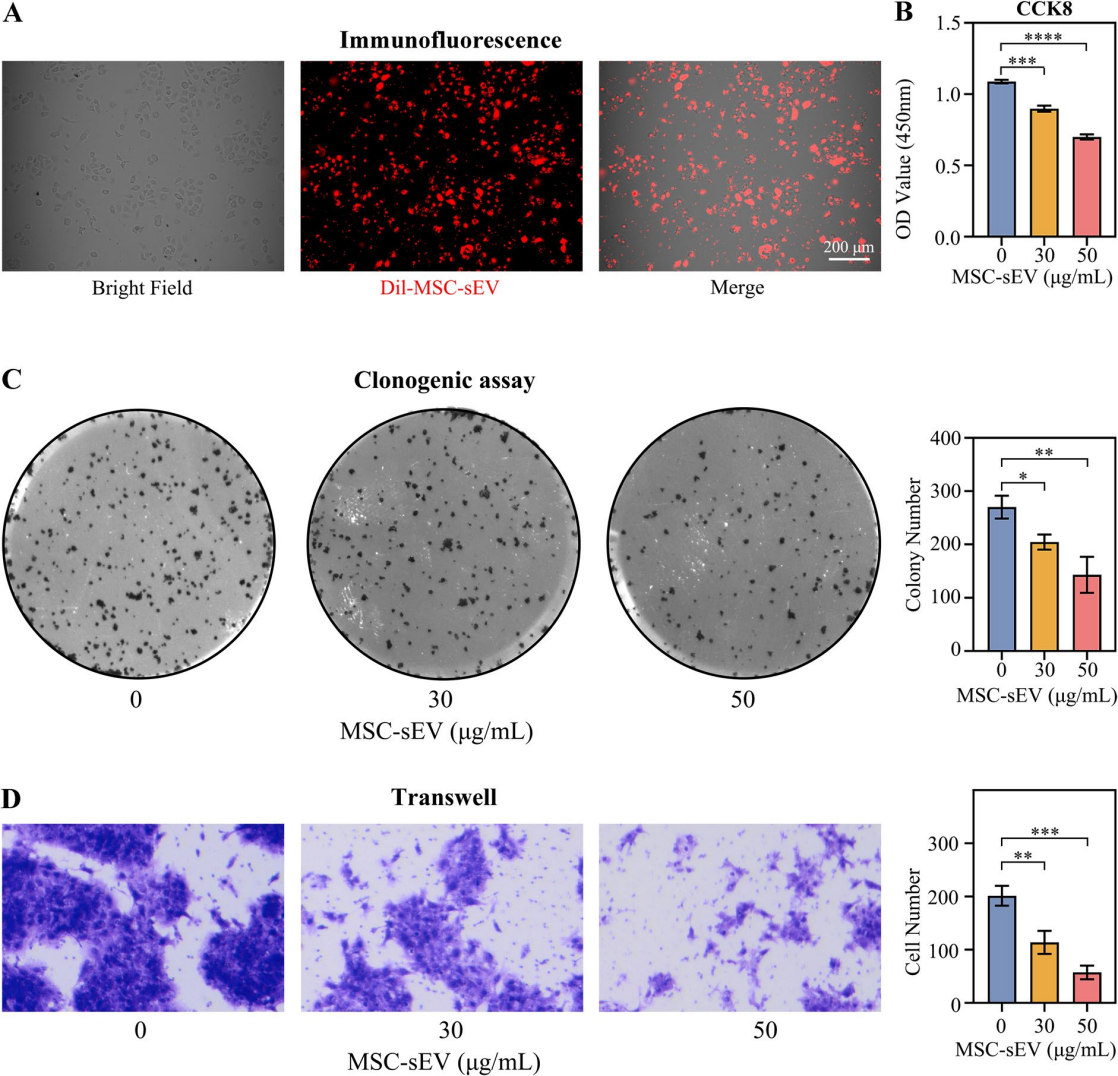
MSC-sEV are absorbed by CaSki cells and inhibited cell viability, colony formation and migration of CaSki cell. **A** Immunofluorescence assay showed CaSki cell uptake Dil (red fluorescence) labeled MSC-sEV (Scale bar, 200 μm). **B** Cell viability of CaSki cells treated with MSC-sEV, OD value in 450 nm were measured. **C** Colony formation ability of MSC-sEV on CaSki cells. **D** Migration ability of MSC-sEV on CaSki cells. *p < 0.05, **p < 0.01, ***p < 0.001, ****p < 0.0001

MSC-sEV labeled with Dil dye were co-cultured with CaSki cells for 24 h, and fluorescence microscopy revealed overlapping signals, indicating CaSki cell uptake of MSC-sEV (Fig. 2A). Next, CaSki cells were treated with various MSC-sEV concentrations (0, 30, 50 μg/mL) to assess cell viability, colony formation, and invasion. Figure 2B showed significantly reduced OD values at 450 nm in the 30 μg/mL group (*p* < 0.001) and further reduction in the 50 μg/mL group (*p* < 0.0001) compared to the 0 μg/mL group. Colony formation assay demonstrated a significant decrease in cell cluster number in the 30 μg/mL group (*p* < 0.05) and a more significant decrease in the 50 μg/mL group (*p* < 0.01) compared to the 0 μg/mL group (Fig. 2C. Transwell assay showed a significant reduction in migrated cells in the 30 μg/mL group (*p* < 0.01) and further reduction in the 50 μg/mL group (*p* < 0.001) compared to the 0 μg/mL group (Fig. 2D).

**Fig. 3:**
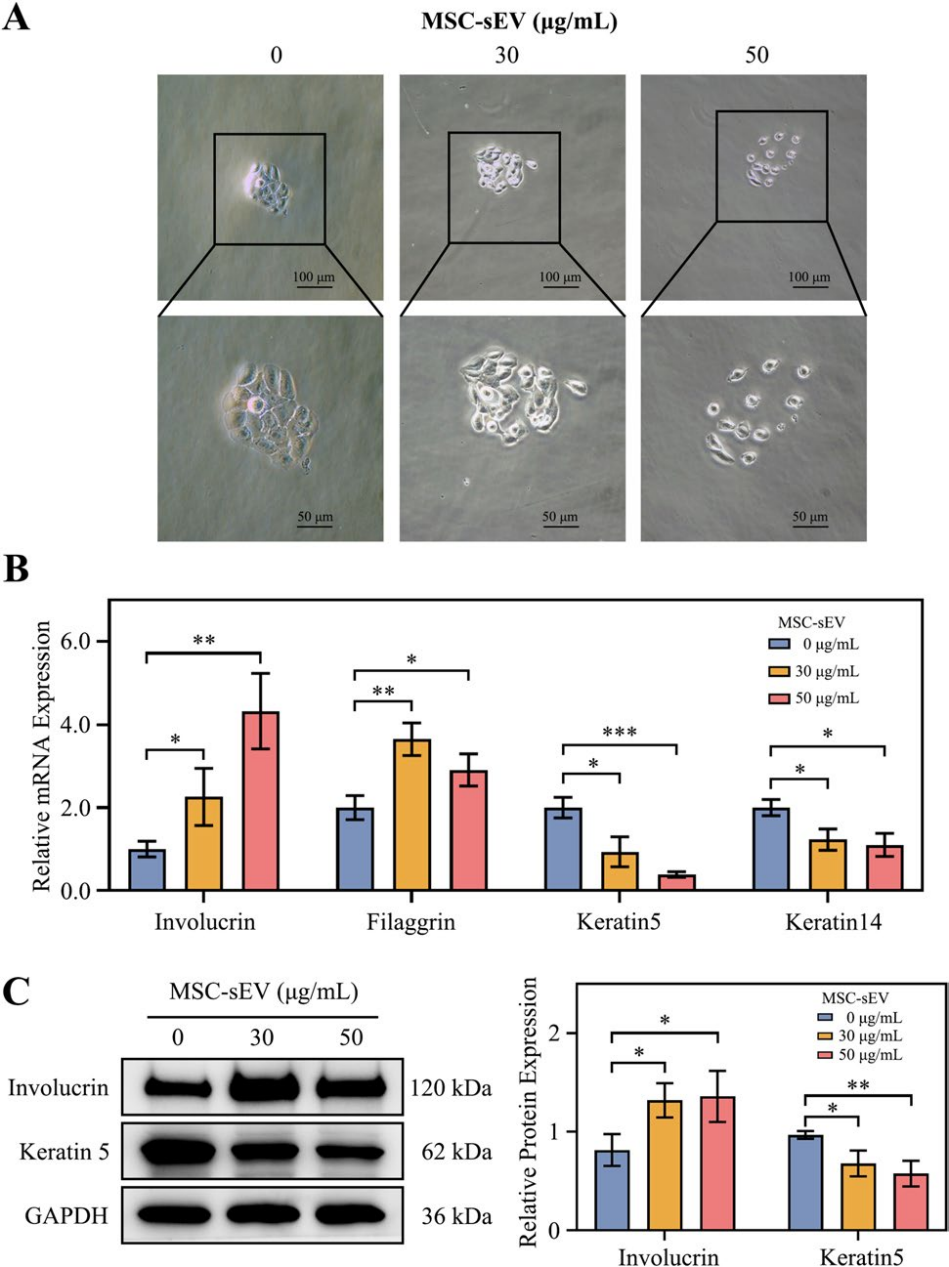
MSC-sEV increased the distance between CaSki cells and promoted squamous differentiation level. **A** Picture of cell colony with different concentration of MSC-sEV (Scale bar, 100 or 50 μm). **B** Relative mRNA expression level of granular layers cell marker (involucrin and filaggrin) and basal cell marker (keratin 5 and keratin 14) after treated with MSC-sEV. **C** The protein expression of involucrin, keratin 5 and β-Actin in CaSki cells after treated with MSC-sEV. *p < 0.05, **p < 0.01, ***p < 0.001.

Overall, MSC-sEV inhibited growth, colony formation, and migration of CaSki cells.

### MSC-sEV promote squamous differentiation level

In the colony formation assay, MSC-sEV absence resulted in tightly connected normal cell clusters. However, addition of MSC-sEV led to increased loosening of cell–cell connections, which intensified with higher MSC-sEV concentrations (Fig. 3A).

In normal cervical tissue, basal cells undergo sequential differentiation into spinous cells, granular cells, and keratinized squamous epithelial cells, known as squamous differentiation. In cervical cancer tissue, cell–cell connections are tight, impairing squamous differentiation. To assess the effect of MSC-sEV on squamous differentiation in CaSki cells, we examined the expression of basal cell markers keratin 5 and keratin 14, as well as granular cells markers filaggrin and involucrin. Results showed MSC-sEV up regulated filaggrin (*p* < 0.05) and involucrin (*p* < 0.05) expression in both concentrations, while downregulating mRNA levels of keratin 5 (*p* < 0.05) and keratin 14 (*p* < 0.05), markers of basal cells. This suggests that MSC-sEV could promote squamous differentiation of CaSki cells (Fig. 3B). And WB showed similar results that MSC-sEV could up regulated involucrin while down regulate keratin 5 (Fig. 3C).

### Jagged1 contained in MSC-sEV activate NOTCH signaling pathway in cervical cancer cells.

The differentiation of basal cells into squamous epithelium requires activation of the NOTCH pathway, while HPV infection inhibits activation of the NOTCH pathway. In order to explore how MSC-sEV promote cervical cancer squamous differentiation, we detected the effects of MSC-sEV on the NOTCH pathway.

**Fig. 4:**
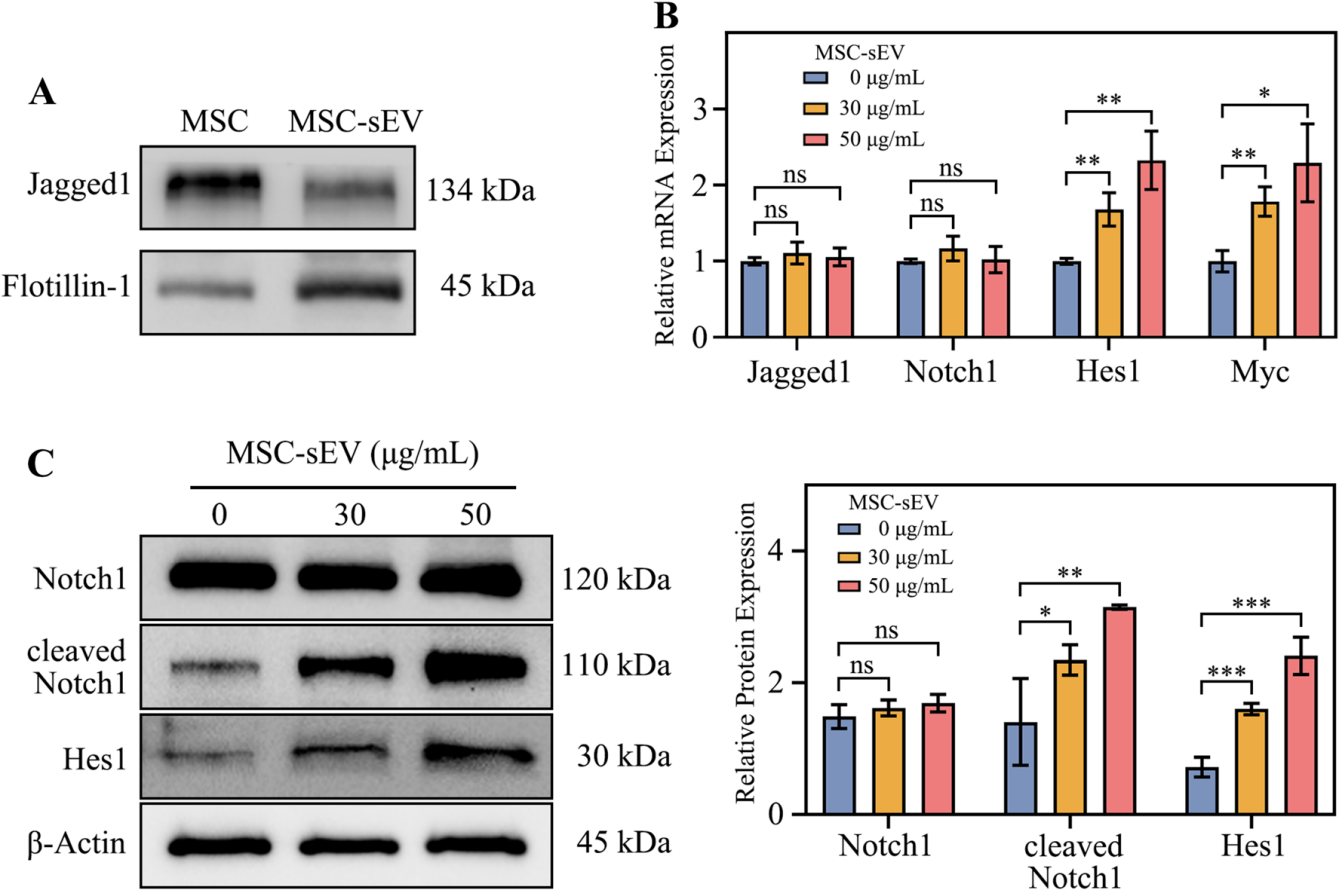
Jagged1 on MSC-sEV activated NOTCH pathway in CaSki cells. **A** The protein expression of jagged1 and flotillin-1 in MSC and MSC-sEV. **B** The mRNA expression of NOTCH pathway molecules (jagged1, notch1, hes1 and myc) after treated with MSC-sEV for 48 h. **C** The protein expression of NOTCH pathway molecules (notch1, cleaved nothc1, hes1 and β-Actin) after treated with MSC-sEV for 72 h. n.s. (no significance), *p < 0.05, **p < 0.01, ***p < 0.001

It has been reported that MSC-sEV contain jagged1 protein, and we also confirmed this (Gonzalez-King et al. 2017). WB results showed that jagged1 is present in both MSC and MSC-sEV (Fig. 4A). We detected the mRNA levels of jagged1, notch1 and the downstream molecules of NOTCH pathway hes1 and myc in cervical cancer cells. The results showed that MSC-sEV had no significant effect on the mRNA levels of jagged1 and notch1, but significantly increased the mRNA levels of hes1 and myc indicating that MSC-sEV activated the transcription of notch downstream molecules (Fig. 4B). We also detected the jagged1, notch1, cleaved notch1 and hes1 proteins (Fig. 4C). The results found that MSC-sEV could significantly increase the protein content of cleaved notch1 and hes1, indicating that MSC-sEV can promote notch1 cleavage, increase Notch1 intracellular domain (NICD) nuclear translocation, and further promote downstream hes1 protein expression.

In summary, our results demonstrate that MSC-sEV can activate the NOTCH pathway and downstream molecule transcription and expression in CaSki cells through the jagged1 on MSC-sEV.

### MSC-sEV activate NOTCH signaling pathway and promote proliferation of HaCaT cells.

Our data shows that MSC-sEV could inhibit the growth, proliferation and migration abilities of CaSki cells by promoting squamous differentiation through activating the NOTCH pathway. However, it is unclear that whether MSC-sEV have the same effects on more differentiated cells. Therefore, we explored the effects of MSC-sEV on human keratinocyte HaCaT cells with higher differentiation levels, to investigate whether CaSki cells should be promoted to differentiate into keratinized cells.

**Fig. 5:**
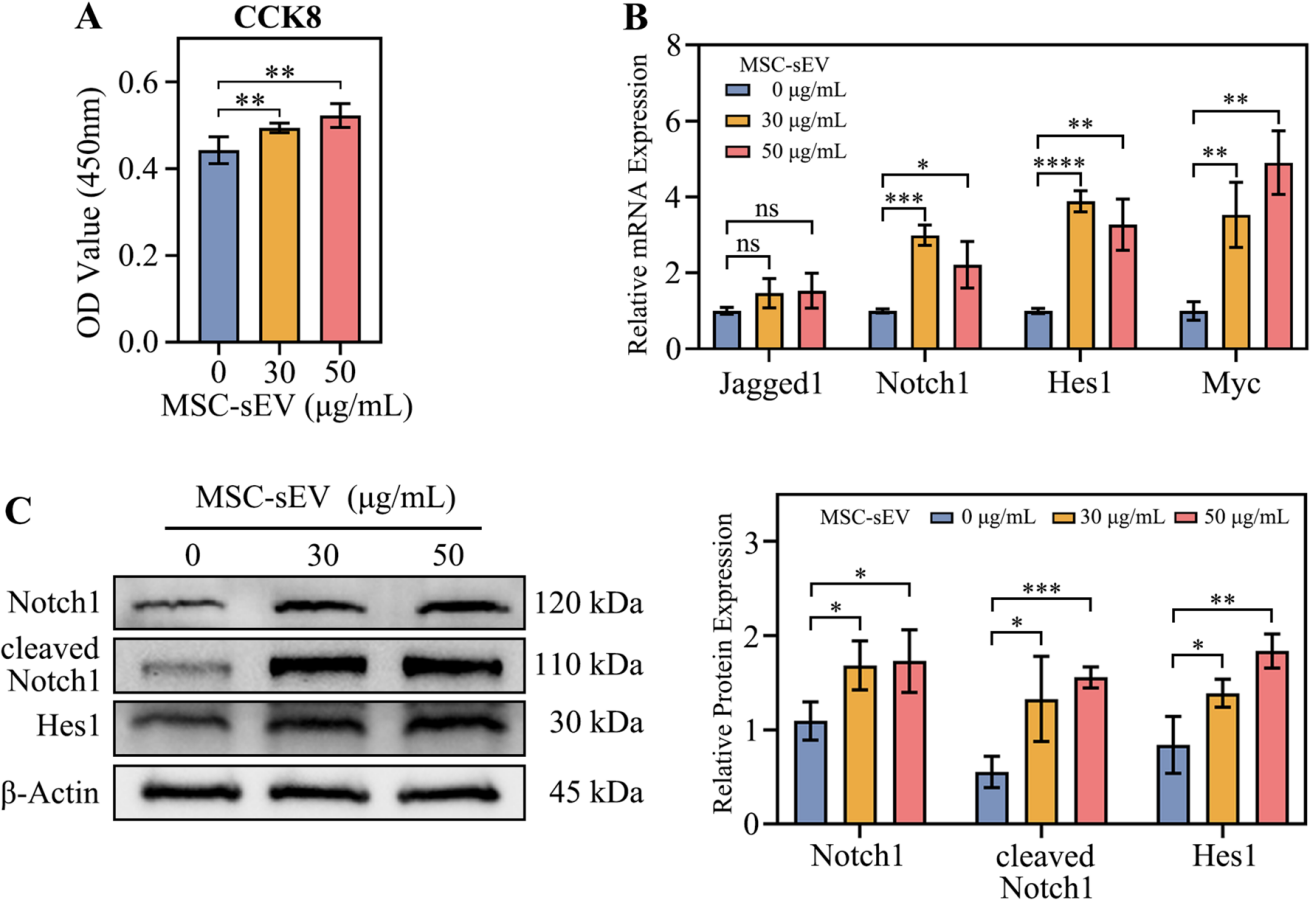
MSC-sEV promoted HaCaT cell viability and activated NOTCH pathway. **A** Cell viability of CaSki cells treated with MSC-sEV, OD value in 450 nm were measured. **B** The mRNA expression of NOTCH pathway molecules (jagged1, notch1, hes1 and myc) after treated with MSC-sEV for 48 h. **C** The protein expression of NOTCH pathway molecules (notch1, cleaved nothc1, hes1 and β-Actin) after treated with MSC-sEV for 72 h. n.s. (no significance), *p < 0.05, **p < 0.01, ***p < 0.001, ****p < 0.0001

We detected the effects of MSC-sEV on HaCaT cell viability. The results showed that MSC-sEV can increase HaCaT cells viability (Fig. 5A). We detected the mRNA levels of jagged1, notch1 and the downstream molecules hes1 and myc. The results showed that MSC-sEV had no significant effect on jagged1 mRNA levels, but significantly increased the mRNA levels of notch1, hes1 and myc, indicating that MSC-sEV can increase notch1 expression and activate transcription of notch downstream molecules (Fig. 5B). We detected jagged1, notch1, cleaved notch1 and hes1 proteins. The results found that MSC-sEV significantly increased the protein levels of notch, cleaved notch1 and hes1, indicating that MSC-sEV can increase notch1 protein content, promote notch1 cleavage, increase NICD nuclear translocation, and further promote downstream hes1 protein expression (Fig. 5C).

## Discussion

Cervical cancer caused by persistent HPV infection is an important threat to women's health. HPV infection can damage the differentiation ability of cervical basal cells, leading to their carcinogenesis and malignant proliferation (White 2019). MSC-sEV have shown great potential in cancer treatment, but their biological effects and specific mechanisms on HPV cervical cancer cells are still unclear. In this study, we found that the Jagged1 molecule contained in MSC-sEV can activate the NOTCH pathway in cervical cancer cells, thereby promoting squamous differentiation of cervical cancer cells and inhibiting their growth and proliferation. In addition, we also explored the effects of MSC-sEV on human keratinocyte HaCaT cells with higher differentiation levels, and found that MSC-sEV can also activate the NOTCH pathway in HaCaT cells, but promote HaCaT cell viability.

Since the squamous differentiation level of squamous cell carcinomas is generally negatively correlated with tumor malignancy, this has led to the concept of differentiation therapy (de Thé 2018). Some studies have achieved certain results in treating precancerous lesions and cervical cancer caused by HPV infection through promoting differentiation. All-trans retinoic acid (ATRA) can inhibit the proliferation of squamous cell carcinoma in non-melanoma skin cancer[15]. Sorafenib promotes the differentiation of skin squamous cell carcinoma SCC by inhibiting the B-Raf/Mek/Erk pathway, and inhibits the growth of its tumor tissue (Cheepala et al. 2009). Our study shows that MSC-sEV can activate the NOTCH pathway in cervical cancer cells, promote their squamous differentiation, and inhibit their growth, proliferation and migration abilities, providing new ideas for the application of differentiation therapy in cervical cancer.

The NOTCH pathway plays an important role in cell differentiation. NOTCH1-4 are transmembrane proteins that can be activated by interacting with Jagged or DLL proteins. After that, metalloproteinase and γ-secretase cleave the NOTCH protein, and the resulting NICD enters the nucleus, forms a complex with RBP-J protein, relieves RBP-J's inhibitory effect, and activates downstream protein transcription (Lefort and Dotto 2004). The HPV E6 protein can bind to MAML1 and prevent activation of the NOTCH pathway (Zanier et al. 2013). Our study demonstrates that MSC-sEV can carry Jagged 1 to activate the NOTCH pathway in cervical cancer cells, promoting their squamous differentiation and inhibiting their growth, proliferation and migration abilities. But it still needs more verification to what extent the E6 protein can hinder the activation of the NOTCH pathway by increased cleaved Notch1.

Compared with HPV-negative C33a cells and primary keratinocytes, NOTCH1 is downregulated in HPV-positive cervical cancer cell lines (such as CaSki) (Zagouras et al. 1995). In primary keratinocytes, inhibiting the NOTCH pathway and activating the Ras pathway can lead to the formation of cancer cells (Rothenberg and Ellisen 2012). The above evidence suggests that NOTCH1 may be a tumor suppressor. We did not observe a significant effect of MSC-sEV on NOTCH1 expression in CaSki, but NOTCH1 expression was significantly upregulated in HaCaT, which need further study.

Many studies have reported that MSC-sEV have different effects on different tumors. MSC-sEV can promote or inhibit VEGF expression through p38/MAPK, mTOR/HIF-1α, NF-κB and other pathways, thereby promoting or inhibiting angiogenesis (Weng et al. 2021). MSC-sEV can promote tumor growth and metastasis through ERK, WNT, PTEN and other pathways (Vakhshiteh et al. 2019). MSC-sEV can inhibit tumor progression by activating apoptosis, blocking the cell cycle and activating immune responses (Wu et al. 2022). Li et al. found that miR-142-3p in vesicles from bone marrow-derived MSC can downregulate Numb and thus activate Notch to promote colon cancer stem cell phenotypes (Li and Li 2018). Our research elucidates mechanisms underlying the inhibitory effect of MSC-sEV on cervical cancer and suggests potential for differentiation therapy.

Similar to CaSki cells, our results show that MSC-sEV could activate NOTCH pathway activation and downstream molecule transcription and expression in HaCaT cells. However, different from CaSki cells, in HaCaT cells, MSC-sEV can increase the expression of notch1 and promote HaCaT cell viability. Several studies have found that MSC-sEV can promote wound healing and enhance the wound healing ability of HaCaT cells. These findings indicate that the phenomenon of MSC-sEV inhibiting CaSki growth and proliferation by promoting squamous differentiation may only exist to a certain degree of differentiation. After CaSki cells differentiate into keratinocytes, MSC-sEV may promote their growth and proliferation.

In our study, there are still some areas that need more research and evidence. We found that MSC-sEV inhibit cervical cancer by promoting cervical cancer squamous differentiation, but the extent to which cervical cancer cells need to differentiate remains to be further studied. We found that MSC-sEV have a promoting effect on HaCaT cells, indicating that unlimited promotion of squamous differentiation by MSC-sEV may not be beneficial to inhibiting cervical cancer, at least to some extent. In addition, HPV-encoded E6 and E7 proteins can damage squamous differentiation, and the direct effects of MSC-sEV on HPV and its encoded oncoproteins also require further study. From HPV infection, cervical intraepithelial neoplasia, to eventual cervical cancer is a long process, and it is still unclear at which stage intervention with MSC-sEV is the most effective, requiring more research. In addition, many studies have focused on MSC-sEV exerting biological effects by inhibiting their target genes with carried microRNAs, while MSC-sEV contain about 900 microRNAs. Therefore, whether and how MSC-sEV affect HPV and cervical cancer cell differentiation through their carried microRNAs also requires more research.

The treatment of CIN and cervical cancer found during pregnancy is a very tricky clinical problem, whether to continue observation or terminate the pregnancy directly needs to comprehensively consider the gestational age and staging of CIN and cervical cancer. In this study, we used sEVs secreted by MSC derived from umbilical cord, and the large number of MSC existing in umbilical cord during pregnancy can also secrete a lot of MSC-sEV, which may promote squamous differentiation of cervical cancer and slow the progression of cervical cancer. But whether clinical decisions should be adjusted still requires more rigorous in vivo experiments and evidence-based medical evidence.

## Conclusion

In summary, we successfully extracted human umbilical cord mesenchymal stem cells and their secreted small extracellular vesicles. Then, we found that MSC-sEV could inhibit the proliferation and migration ability of cervical cancer cells. And then, we found that this inhibition may be due to the Jagged1 molecules on the surface of MSC-sEV promoting the activation of NOTCH pathway and promoting the squamous differentiation of cervical cancer cells. We also observed MSC-sEV could activate NOTCH pathway in HaCaT cells, but it can promote the cell viability of HaCaT cells. These findings reveal the possible mechanism of MSC-sEV in treating cervical cancer.

## Supplementary Information

Below is the link to the electronic supplementary material.

Supplementary file1 (DOCX 1687 KB)

Supplementary file2 (DOCX 17 KB)

## Data Availability

All data generated or analyzed in this study are included in this published article.

## Abbreviations

CCK8: Cell counting kit-8
CIN: Cervical intraepithelial neoplasia
EMT: Epithelial-mesenchymal transition
HPV: Human papillomavirus
hucMSC: Human umbilical cord mesenchymal stem cells
ISCT: International Society for Cellular Therapy
MSC: Mesenchymal stem cells
MSC-sEV: Mesenchymal stem cell-derived small extracellular vesicles
NC: Negative control
NTA: Nanoparticle tracking analysis
qPCR: Real-time quantitative polymerase chain reaction
TEM: Transmission electron microscopy
WB: Western Blot
