# Supplementary material for: Jagged1 contained in MSC-derived small extracellular vesicles promotes squamous differentiation of cervical cancer by activating NOTCH pathway
Source: J Cancer Res Clin Oncol. 2023 Nov 23;149(20):18093–102. doi: 10.1007/s00432-023-05495-3 (PMC10725371; doi:10.1007/s00432-023-05495-3)

**Jagged1 contained in MSC-Derived Small Extracellular Vesicles Promotes Squamous Differentiation of Cervical Cancer by Activating NOTCH Pathway**

Weizhao Li^1,2#^, Xunzhi Zhang^3#^, Tianshun Gao^1#^, Lixiang Liu^1^, Chi Zhang^1^, Huan Yang^1^, Jiayuan Xie^1^, Wei Pan^1^, David YB Deng^1*^, Changlin Zhang^1,2*^, Tian Li^1,2*^

^1^ Department of Gynecology, Pelvic Floor disorders Center, Scientific Research Center, The Seventh Affiliated Hospital of Sun Yat-sen University, Shenzhen, China

^2^ Shenzhen Key Laboratory of Chinese Medicine Active substance screening and Translational Research, Shenzhen, China

^3^ College of Life Sciences and Oceanography, Shenzhen University, Shenzhen, China

***Correspondence:**

David YB Deng, dengyub@mail.sysu.edu.cn, Changlin Zhang, zhangchanglin@sysush.com and Tian Li, litian@sysush.com.

#These authors contributed equally to this work and share first authorship.

**Figure legend**

**Supplementary Figure 1. Identification of hucMSC.** (A) Wharton’s jelly and MSC attach on plastic. (B) Alizarin red S staining the MSC after osteogenic induction. (C) Oil-red O staining the MSC after adipogenic induction. (D-F) Flow cytometry analysis dectected the positive surface marker of MSC. (G-K) Flow cytometry analysis dectected the negative surface marker of MSC.

**Supplementary Figure 2. Graphical Abstract.**

**Supplementary Figure 1**


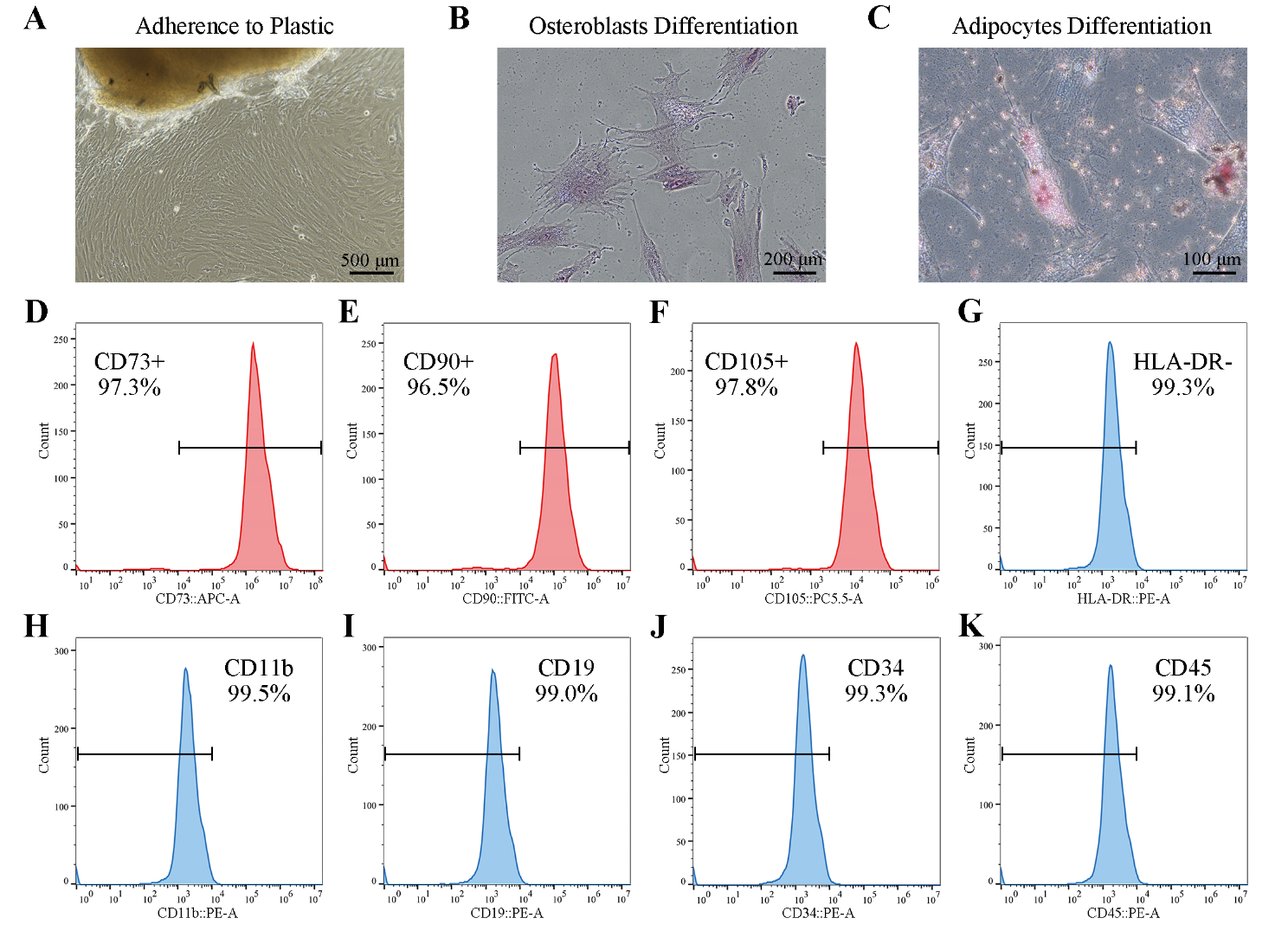


**Supplementary Figure 2**


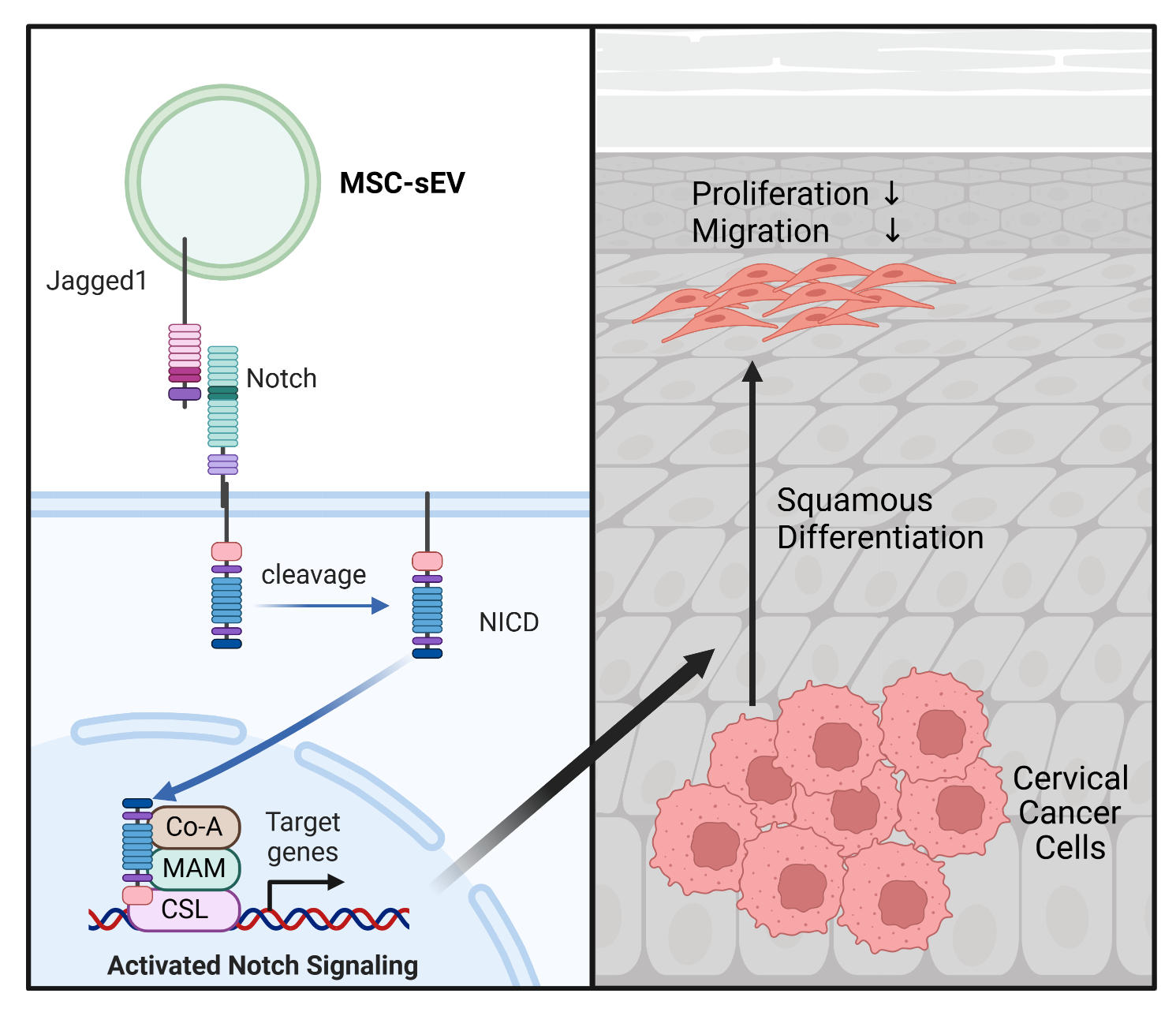

Supplement: Supplementary file 1 — Supplementary file1 (DOCX 1687 KB) [file 432_2023_5495_MOESM1_ESM.docx]
