# Supplementary material for: Jagged1 contained in MSC-derived small extracellular vesicles promotes squamous differentiation of cervical cancer by activating NOTCH pathway
Source: J Cancer Res Clin Oncol. 2023 Nov 23;149(20):18093–102. doi: 10.1007/s00432-023-05495-3 (PMC10725371; doi:10.1007/s00432-023-05495-3)
Supplement: Supplementary file 2 — Supplementary file2 (DOCX 17 KB) [file 432_2023_5495_MOESM2_ESM.docx]

**Jagged1 contained in MSC-Derived Small Extracellular Vesicles Promotes Squamous Differentiation of Cervical Cancer by Activating NOTCH Pathway**

Weizhao Li^1,2#^, Xunzhi Zhang^3#^, Tianshun Gao^1#^, Lixiang Liu^1^, Chi Zhang^1^, Huan Yang^1^, Jiayuan Xie^1^, Wei Pan^1^, David YB Deng^1*^, Changlin Zhang^1,2*^, Tian Li^1,2*^

^1^ Department of Gynecology, Pelvic Floor disorders Center, Scientific Research Center, The Seventh Affiliated Hospital of Sun Yat-sen University, Shenzhen, China

^2^ Shenzhen Key Laboratory of Chinese Medicine Active substance screening and Translational Research, Shenzhen, China

^3^ College of Life Sciences and Oceanography, Shenzhen University, Shenzhen, China

***Correspondence:**

David YB Deng, dengyub@mail.sysu.edu.cn, Changlin Zhang, zhangchanglin@sysush.com and Tian Li, litian@sysush.com.

#These authors contributed equally to this work and share first authorship.

**Supplementary Table 1. Antibody list.**

| **Antibody Name** | **Brand** | **Production Place** | **Product Code** | **Host species** | **Molecular Weight** | **Dilutions** |
| --- | --- | --- | --- | --- | --- | --- |
| CD9 | Proteintech | China | 60232-1-Ig | Mouse | 25 kDa | 1:2,000 |
| Tsg101 | Proteintech | China | 28283-1-AP | Rabbit | 44 kDa | 1:2,000 |
| Calnexin | Proteintech | China | 10427-2-AP | Rabbit | 90 kDa | 1:20,000 |
| Involucrin | Proteintech | China | 28462-1-AP | Rabbit | 120 kDa | 1:4,000 |
| Keratin 5 | Proteintech | China | 28506-1-AP | Rabbit | 63 kDa | 1:6,000 |
| Jagged1 | Huaan Bio | China | ET1702-63 | Rabbit | 134 kDa | 1:1,000 |
| Flotillin-1 | PTM Bio | China | PTM-5369 | Mouse | 45 kDa | 1:1,000 |
| Notch1 | SAB | USA | #54258 | Rabbit | 120 kDa | 1:2,000 |
| Cleaved Notch1 | CST | USA | #4147 | Rabbit | 110 kDa | 1:1,000 |
| Hes1 | Huaan Bio | China | ET1610-97 | Rabbit | 30 kDa | 1:1,000 |
| β-Actin | Proteintech | China | 81115-1-RR | Rabbit | 42 kDa | 1:50,000 |
| HRP Goat Anti-Mouse IgG | Proteintech | China | SA00001-1 | Goat | - | 1:10,000 |
| HRP Goat Anti-Rabbit IgG | Proteintech | China | SA00001-2 | Goat | - | 1:10,000 |

**Supplementary Table 2. Nucleotide sequence of the primer pairs used for the genes in qPCR analysis.**

| **Primer Name** | | **Sequence (5’ to 3’)** |
| --- | --- | --- |
| β-Actin | Forward: | CATTGCTGACAGGATGCAGAAGG |
|  | Reverse: | TGCTGGAAGGTGGACAGTGAGG |
| Involucrin | Forward: | GGTCCAAGACATTCAACCAGCC |
|  | Reverse: | TCTGGACACTGCGGGTGGTTAT |
| Filaggrin | Forward: | GCTGAAGGAACTTCTGGAAAAGG |
|  | Reverse: | GTTGTGGTCTATATCCAAGTGATC |
| Keratin 5 | Forward: | GCTGCCTACATGAACAAGGTGG |
|  | Reverse: | ATGGAGAGGACCACTGAGGTGT |
| Keratin 14 | Forward: | TGCCGAGGAATGGTTCTTCACC |
|  | Reverse: | GCAGCTCAATCTCCAGGTTCTG |
| Jagged1 | Forward: | TGCTACAACCGTGCCAGTGACT |
|  | Reverse: | TCAGGTGTGTCGTTGGAAGCCA |
| Notch1 | Forward: | GGTGAACTGCTCTGAGGAGATC |
|  | Reverse: | GGATTGCAGTCGTCCACGTTGA |
| Hes1 | Forward: | GGAAATGACAGTGAAGCACCTCC |
|  | Reverse: | GAAGCGGGTCACCTCGTTCATG |
| Myc | Forward: | CCTGGTGCTCCATGAGGAGAC |
|  | Reverse: | CAGACTCTGACCTTTTGCCAGG |
